# Among Trauma Patients, Younger Men with Ventilator-Associated Pneumonia Have Worse Outcomes Compared to Older Men—An Exploratory Study

**DOI:** 10.3390/healthcare7020067

**Published:** 2019-04-30

**Authors:** Duraid Younan, Sarah J. Delozier, Nathaniel McQuay, John Adamski, Aisha Violette, Andrew Loudon, Jeffrey Ustin, Regan Berg, Glen Tinkoff, Matthew L. Moorman, UHRISES Research Consortium

**Affiliations:** 1 Department of Surgery, Division of Acute Care Surgery, University Hospitals Cleveland, 11100 Euclid Avenue, 3700 Bolwell, Cleveland, OH 44106, USA; nathaniel.mcquay@uhhospitals.org (N.M.); john.adamski@uhhospitals.org (J.A.); aisha.violette@uhhospitals.org (A.V.); andrew.loudon@uhhospitals.org (A.L.); jeffrey.ustin@uhhospitals.org (J.U.); regan.berg@uhhospitals.org (R.B.); glen.tinkoff@uhhospitals.org (G.T.); matthew.moorman@uhhospitals.org (M.L.M.); 2 Center for Clinical Research, University Hospitals Cleveland, Cleveland, OH 44106, USA; sarah.delozier@uhhospitals.org; 3 University Hospitals Research in Surgical outcomes & Effectiveness center, Department of Surgery, University Hospitals Cleveland, Cleveland, OH 44106, USA

**Keywords:** Trauma, ventilator-associated pneumonia, outcome

## Abstract

Background: Ventilator-associated pneumonia is associated with significant morbidity. Although the association of gender with outcomes in trauma patients has been debated for years, recently, certain authors have demonstrated a difference. We sought to compare the outcomes of younger men and women to older men and women, among critically ill trauma patients with ventilator-associated pneumonia (VAP). Methods: We reviewed our trauma data base for trauma patients with ventilator-associated pneumonia admitted to our trauma intensive care unit between January 2016 and June 2018. Data collected included demographics, injury mechanism and severity (ISS), admission vital signs and laboratory data and outcome measures including hospital length of stay, ICU stay and survival. Patients were also divided into younger (<50) and older (≥50) to account for hormonal status. Linear regression and binary logistic regression models were performed to compare younger men to older men and younger women to older women, and to examine the association between gender and hospital length of stay (LOS), ICU stay (ICUS), and survival. Results: Forty-five trauma patients admitted to our trauma intensive care unit during the study period (January 2016 to August 2018) had ventilator-associated pneumonia. The average age was 58.9 ± 19.6 years with mean ISS of 18.2 ± 9.8. There were 32 (71.1%) men, 27 (60.0%) White, and 41 (91.1%) had blunt trauma. Mean ICU stay was 14.9 ± 11.4 days and mean total hospital length of stay (LOS) was 21.5 ± 14.6 days. Younger men with VAP had longer hospital LOS 28.6 ± 17.1 days compared to older men 16.7 ± 6.6 days, (*p* < 0.001) and longer intensive care unit stay 21.6 ± 15.6 days compared to older men 11.9 ± 7.3 days (*p* = 0.02), there was no significant difference in injury severity (ISS was 22.2 ± 8.4 vs. 17 ± 8, *p* = 0.09). Conclusions: Among trauma patients with VAP, younger men had longer hospital length of stay and a trend towards longer ICU stay. Further research should focus on the mechanisms behind this difference in outcome using a larger database.

## 1. Introduction

Trauma remains the leading cause of death in the United States among individuals aged 1 to 46 years [1]. Trauma and injury result in a systemic body response characterized by acute nonspecific immune response that can be associated with weakened immunity and decreased resistance to infection. This can result in damage to multiple organs from the initial cascade of inflammation aggravated by subsequent sepsis for which the body has become vulnerable to [2]. 

Ventilator-associated pneumonia (VAP) remains the most common nosocomial infection in patients with trauma requiring prolonged mechanical ventilation [3]. Multiple authors have found ventilator-associated pneumonias to be a major cause of morbidity and mortality in different patient populations [4,5,6]; it is associated with prolonged hospital and intensive care unit lengths of stay, days on mechanical ventilation and an attributable mortality of approximately 13% [7]. 

Sex hormones have been found to be associated with outcomes before; after burn injury: estrogen is known to have an inhibitory role on the inflammatory response [8] and antecedent ovariectomy was found to improve survival [9] although researchers have not agreed on the gender-associated difference in outcome in trauma patients [10,11]. 

We hypothesized that injured men and women with VAP have different outcomes based on their age. We sought to compare the outcomes of younger (<50) men and women to older (>50) men and women, among critically ill trauma patients with ventilator-associated pneumonia (VAP) with a view to clinical utilization of this association.

## 2. Materials and Methods

### 2.1. Patient Selection and Variable Definition

After obtaining the Institutional Review Board (IRB) approval, a retrospective analysis of trauma patients admitted to our level 1 trauma center’s intensive care unit between January 2016 and August 2018 with ventilator-associated pneumonia was conducted using the trauma database. The patients came to the hospital as trauma system activations and required admission to the trauma ICU per the judgment of the trauma surgeon on duty.

### 2.2. Inclusion and Exclusion Criteria

**Figure 1:**
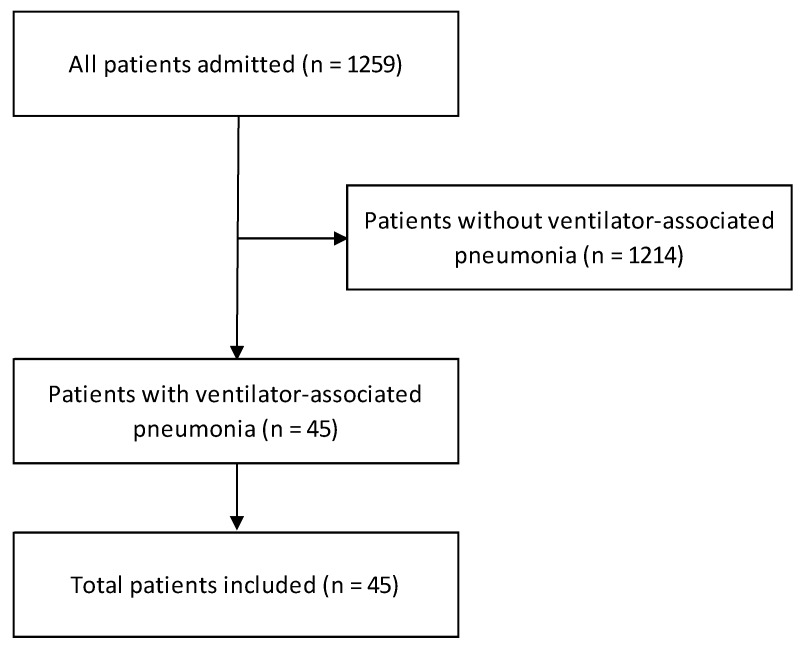
Flowchart of patient inclusion criteria.

All adult trauma patients who were admitted to the trauma ICU over the study period were considered for inclusion; minors (less than 18 years of age) were excluded from the study (Figure 1). Data collected included demographics (age, gender, and race), mechanism of injury (blunt, penetrating), injury severity (injury severity score “ISS”), presence of ventilator-associated pneumonia (VAP), admission vital signs and laboratory data including International Normalization Ratio (INR), hematocrit, platelet count, creatinine, and blood transfusion data (red blood cells, fresh frozen plasma, and platelets). Clinical endpoints included disposition (home, rehabilitation hospital, hospice, and skilled nursing facility), hospital length of stay (total number of days as in-patient), ICU stay (total number of days spent in the intensive care), and death.

### 2.3. Statistical Analysis

Demographics and clinical characteristics were summarized using descriptive statistics (mean and standard deviation for continuous variables; frequency and percentage for categorical variables). Patients were divided into younger (<50 years of age) and older (≥50 years). The cutoff age was chosen to account for hormonal status changes in women and was used for men too. 

Analyses compared younger to older men and younger to older women, adjusting for injury severity score (ISS). All analyses were conducted comparing outcomes (length of stay, ICU days, survival, and tracheostomy) for patients with ventilator-associated pneumonia (VAP), using the predictor variables of gender and age (<50, 50+). *t*-tests were used to examine ISS as a function of age and sex. Of particular interest was identifying potential age by gender differences or trends. Pearson and point biserial correlations were used to identify predictive factors for these outcomes. Multiple regression and binary logistic regression models were used to examine primary outcomes while controlling for the effects of ISS. Regressions were fit controlling for ISS and including age (<50, 50+), gender, and the age by gender interaction were included as predictor variables using the enter method and an indicator contrast. Follow-up regressions were then run separately stratified by age and by gender to examine potential age and gender differences in greater detail. All analyses were two-tailed, and *p* ≤ 0.05 was considered significant. All analyses were run using SPSS V.25.0 (Armonk, NY: IBM Corp.) 

## 3. Results 

Forty-five trauma patients admitted to our trauma intensive care unit during the study period had ventilator-associated pneumonia. The average age was 58.9 ± 19.6 years with mean ISS of 18.2 ± 9.8. There were 32 (71.1%) men, 27 (60.0%) White were, and 41 (91.1%) had blunt trauma. Mean ICU stay was 14.9 ± 11.4 days and mean total hospital length of stay (LOS) was 21.5 ± 14.6 days (Table 1).

Younger men with VAP had longer hospital LOS 28.6 ± 17.1 days compared to older men 16.7 ± 6.6 days, (*p* < 0.001) and longer intensive care unit stay 21.6 ± 15.6 days compared to older men 11.9 ± 7.3 days (*p* = 0.02), there was no significant difference in injury severity (ISS was 22.2 ± 8.4 vs. 17 ± 8, *p* = 0.09) (Table 2).

Younger women had similar hospital length of stay, ICU stay, and ISS compared to older women. They had a higher hematocrit value at 24 h (42.7 vs. 30.5, *p* = 0.01) (Table 3). Younger adults had similar hospital length of stay (29.14 ± 20.44) compared to older adults (18.06 ± 9.56), *p* = 0.262

LOS was not reliably predicted by age, *p* = 0.416, or sex, *p* = 0.674, and the age by sex interaction was also not significant, *p* = 0.865. Examined separately by sex and age, LOS was greater for younger than older males, *p* = 0.011, but this was not found for females, *p* = 0.624. Sex could not reliably predict LOS for younger adults, *p* = 0.777, or older adults, *p* = 0.157

Number of ICU days was not reliably predicted by sex, *p* = 0.209, or age, *p* = 0.510. The age by sex interaction also was not significant, *p* = 0.137. Examined separately by sex and age, ICU days trended towards longer stays for younger males than older males, *p* = 0.066; ICU days were similar for women of both ages, *p* = 0.330. ICU days were similar between older adults of both sexes, *p* = 0.286, and between younger adults of both sexes, *p* = 0.386. 

Survival was not reliably predicted by sex, *p* = 0.568, age, *p* = 0.678, or the age by sex interaction, *p* = 0.495. Examined separately by sex and age however, age did not reliably predict survival for females, *p* = 0.346, or for males, *p* = 0.418, nor did sex did reliably predict survival for younger adults, *p* = 0.575, or older adults, *p* = 0.572. 

Adjusted for ISS, younger men with VAP had longer LOS (*p* = 0.02), ICU stay trended towards longer stays for younger males but did not reach statistical significance (*p* = 0.06). There was no difference in LOS and ICUS between younger and older women (Table 4).

Injury types (head, chest, and abdomen) were distributed similarly amongst younger and older men for head injuries, *p* = 0.999, and chest injuries, *p* = 0.999, but younger males were more likely to have abdomen injuries than older men, *p* = 0.037. Younger and older females had similar injury rates for head, *p* = 0.559, chest, *p* = 0.999, and abdomen, *p* = 0.231. 

Post-hoc power analyses indicated that for the overall models including the age by sex interaction, two-tailed regression analyses with three predictor variables, alpha ≤ 0.05, resulted in achieved power of 0.57 (survival), 0.27 (tracheostomy), 0.50 (hospital length of stay), and 0.43 (ICU length of stay). For analyses conducted separately by men, power was 0.59 (ICU length of stay), 0.71 (hospital length of stay), 0.03 (tracheotomy), and 0.35 (survival). For analyses conducted separately by women, power was 0.07 (ICU length of stay), 0.34 (hospital length of stay), 0.07 (tracheotomy), and 0.27 (survival).

## 4. Discussion 

We found that injured critically ill younger men with ventilator-associated pneumonia have significantly longer hospital lengths of stay compared to older men, even after adjusting for injury severity. Intensive care unit stay trended towards significance among younger men, this was not significant in females. This finding can have implications on the care of the trauma patients with ventilator-associated pneumonia, and help direct available resources in the future.

Major trauma elicits a strong inflammatory response. Gender differences in the inflammatory response have been demonstrated in the literature; while certain authors attributed the change in X chromosome expression was caused by the cell acid–base balance [12], others attributed it to the different effects of estrogen and testosterone on the immune response caused changes in the innate immune cell responses after injury [13]. Recently, this inflammatory response has been linked to outcomes in critically ill trauma patients [14].

Testosterone has been shown to play a role in many organ systems, it is also known to decrease in older men [15] While gender differences in patients’ outcomes have been reported in patients who suffered from ruptured aortic aneurysms, traumatic head injury and Achilles tendon injury [16,17,18], large studies in trauma patients have not demonstrated a difference in outcome attributed to gender. Gannon et al., in a study of 22,332 trauma patients admitted to trauma centers in Pennsylvania over two years, found that female gender did not affect mortality when stratified for ISS and age, and Magnotti et al., in a retrospective analysis of trauma patients over ten years, found gender not to be associated with mortality [10,11]. Both studies did not look specifically at patients with VAP and did not study different age groups. 

Rello et al., and others found in a multiple logistic regression analysis that male gender was independently associated with the development of VAP [11,19,20], and Sharpe et al. found that females developed less VAP but experienced increased mortality [21]. Other authors have similarly identified gender differences in incidence of pneumonia [22,23,24]. Although testosterone is known to have anti-inflammatory properties and to decrease with age [25], in this study, critically injured men younger than 50 years of age with ventilator-associated pneumonia had significantly longer hospital length of stay than older men regardless of injury severity and had a trend towards longer ICU stay. On the other hand, women younger than 50, compared to those older than 50 years of age, did not have a significantly higher ISS, received similar numbers of packed red blood cells, had similar lactic acid level, did not have differing length of stay in hospital nor in ICU. We did not have enough patients in the less than 50 group to demonstrate a difference in outcomes between the two. To the best of our knowledge, this is the first study identifying differences in outcomes between certain age groups.

While younger men with ventilator-associated pneumonia did not have a higher injury severity or a significant difference in the mechanism of injury when compared to older men, they did have more abdominal injuries. This could have contributed to the longer length of stay and intensive care unit stay noted in these patients. There was no difference noted in injury types between younger and older women.

Although hospitals have in the past reported prolonged periods of decreased ventilator-associated pneumonia rates and even periods free of VAP [26,27], identifying a patient population at risk for worse outcomes (younger men) helps direct the care and available resources to this group in the future and thus improve their outcomes. Larger prospective studies can better evaluate the effects of gender and hormonal status of these patients on outcomes. 

Our clinical study makes the observation that estrogen may be a protective factor when recovering from a VAP. Current laboratory study of estrogen highlights beneficial effects on cytokine release, chemotaxis of neutrophils, expression of heat shock proteins, and restoration of organ function following shock and sepsis [28]. The greatest assumption in our retrospective study is that age and sex correlates to hormone level (estrogen level) in the patient at the time of trauma. A prospective study measuring estrogen level at the time of admission would better evaluate the relationship between length of stay and hormone level. Furthermore, we do not account for the varying level of estrogen at different time of menses, or for estrogen levels associated with obesity/fat store. Our data supports the theory that estrogen may mitigate the inflammatory cascade in VAP, thereby reducing overall length of stay. While men with VAP trended toward a longer ICU length of stay, we cannot support this finding with our underpowered study. We recommend additional research focusing on ICU length of stay in male patients with VAP as an adequately powered study may reach significance. 

Our study should also be appraised in light of its limitations. First, the study is retrospective, performed at a single-center, decreasing the generalizability of our findings. Second, the available data in the trauma database were limited to two and a half years and that resulted in a small sample size. Having a larger size would have enabled us to better evaluate the outcomes of women with ventilator-associated pneumonias of differing age groups and thus better compare them to the outcome of men. Third, other factors unknown to us could have contributed to the difference in outcome between younger and older men with VAP. Fourth, we did not have levels of hormones for these patients, to correlate with findings. Fifth: the hematocrit was significantly different between younger and older women even though the injury severity and transfusion were not different. This can have implications on the care of these of these patients, as oxygen carrying capacity will differ and this can affect ventilator management and weaning.

## 5. Conclusions

Younger men with ventilator-associated pneumonia had longer hospital length of stay and trended towards longer intensive care unit stay despite having an injury severity that was not significantly different. No significant differences in outcomes were identified between younger and older women. Further studies are needed to investigate these findings.

## Figures and Tables

**Table 1:** Demographic, injury, and clinical characteristics of all trauma patients (male and female) with ventilator-associated pneumonia (VAP), *N* = 45.

Demographics	Data
Mean age (Years)	58.9 (19.6)
Race (%)	
White	27 (60.0)
Black	18 (40.0)
Gender, *n* (%)	
Male	32 (71.1)
Female	13 (28.9)
**Injury,***n* (%)	
Blunt	41 (91.1)
Penetrating	4 (8.9)
**Clinical**	
ED Systolic BP	142 ± 32
ED Heart rate	95 ± 21
ED Respiratory rate	20 ± 4
Platelet count at admission	221.4 ± 90
Platelet count at 24 h	176.7 ± 95.4
INR at admission	1.2 ± 0.2
INR at 24 h	1.2 ± 0.3
Hematocrit at admission	35.8 ± 6.1
Hematocrit at 24 h	32 ± 7.3
Creatinine at admission	1.3 ± 1.7
Lactic acid on admission	2.9 ± 1.4
Lactic acid at 24 h	2.0 ± 1.4
Packed red blood cells received first 24 h	2.2 ± 5.1
Packed red blood cells transfused (total)	2.4 ± 5.0
ISS (Injury Severity Score)	18.2 ± 9.8
Tracheostomy, *n* (%)	34 (75.6)
Disposition, *n* (%)	
Home	4 (8.9)
Rehab. hospital	10 (22.2)
Skilled nursing facility (SNF)	19 (42.2)
Hospice	4 (8.9)
Hospital length of stay (days)	21.5 ± 14.6
ICU length of stay	14.9 ± 11.4
Dead, *n* (%)	8 (17.8)

**Table 2:** Demographic, injury, and clinical characteristics of male trauma patients with ventilator-associated pneumonia (VAP).

Variable	Younger Men *N* = 11	Older Men *N* = 21	* p
**Demographics**			
Mean age (Years)	36.9 ± 9.1	64.2 ± 11.6	<0.001 *
Race (%)			0.99
White	6 (54.4)	12 (57.1)	
Black	5 (45.4)	9 (42.9)	
**Injury,***n* (%)			0.11
Blunt	8 (72.7)	20 (95.2)	
Penetrating	3 (27.2)	1 (4.8)	
**Clinical**			
ED Systolic BP	147 ± 32	137 ± 34	0.44
ED Heart rate	89 ± 17	91 ± 19	0.76
ED Respiratory rate	20 ± 5	19 ± 4	0.55
Platelet count at admission	229.1 ± 117.1	219.7 ± 109	0.81
Platelet count at 24 h	170.4 ± 75	191.2 ± 117.2	0.62
INR at admission	1.1 ± 0.2	1.2 ± 0.3	0.26
INR at 24 h	1.4 ± 0.3	1.2 ± 0.3	0.17
Hematocrit at admission	37.1 ± 7.2	35.4 ± 5.4	0.49
Hematocrit at 24 h	33.7 ± 5.6	30.5 ± 8.3	0.29
Creatinine at admission	1.0 ± 0.4	1.1 ± 0.7	0.74
Lactic acid on admission	3.7 ± 1.5	2.7 ± 1.4	0.13
Lactic acid at 24 h	2.5 ± 1.6	2 ± 1.4	0.49
Packed red blood cells received first 24 h	3.9 ± 8.0	2.5 ± 4.5	0.56
Packed red blood cells transfused (total)	4.0 ± 8	2.7 ± 4.4	0.58
ISS (Injury Severity Score)	22.2 ± 8.4	17 ± 8	0.09
Tracheostomy	2 (18.2)	2 (9.5)	0.59
Disposition, *n* (%)			0.94
Home	3 (27.3)	1 (4.8)	
Rehab. hospital	4 (36.4)	4 (19.0)	
Skilled nursing facility (SNF)	2 (18.2)	10 (47.6)	
Hospice	0 (0.0)	2 (9.5)	
Hospital length of stay (days)	28.6 ± 17.1	16.7 ± 6.6	<0.001 *
ICU length of stay	21.6 ± 15.6	11.9 ± 7.3	0.02 *
Dead, *n* (%)	2 (18.2)	4 (19.0)	0.99

* *p*-value is result of *t*-test and chi-squared or Fisher’s exact tests for continuous and categorical variables, respectively.

**Table 3:** Demographic, injury, and clinical characteristics of female trauma patients with ventilator-associated pneumonia (VAP).

Variable	Younger Women *N* = 3	Older Women *N* = 10	* p
**Demographics**			
Mean age (Years)	33.7 ± 11.6	79.5 ± 10.6	<0.001 *
Race (%)			0.20
White	1 (33.3)	8 (80.0)	
Black	2 (66.7)	2 (20.0)	
**Injury,***n* (%)			0.99
Blunt	3 (100.0)	10 (100.0)	
Penetrating	0 (0.0)	0 (0.0)	
**Clinical**			
ED Systolic BP	115 ± 26	155 ± 28	0.07
ED Heart rate	112 ± 24	105 ± 27	0.70
ED Respiratory rate	23 ± 6	19 ± 4	0.27
Platelet count at admission	199.5 ± 50.2	221.3 ± 75.7	0.71
Platelet count at 24 h	183.0 ± 42.4	139.0 ± 54.9	0.35
INR at admission	1.15 ± 0.07	1.13 ± 0.18	0.88
INR at 24 h	1.12 ± 0.14	1.12 ± 0.25	0.99
Hematocrit at admission	41.3 ± 9.3	34.2 ± 5.9	0.18
Hematocrit at 24 h	42.7 ± 8.1	30.5 ± 8.1	0.01 *
Creatinine at admission	0.6 ± 0.5	2.3 ± 3.5	0.53
Lactic acid on admission	2.5 ± 0.0	2.2 ± 1.1	0.78
Lactic acid at 24 h	-	1.20 ± 0.00	-
Packed red blood cells received first 24 h	0.0 ± 0.0	0.5 ± 1.6	0.61
Packed red blood cells transfused (total)	0.0 ± 0.0	0.5 ± 1.6	0.61
ISS (Injury Severity Score)	21.0 ± 25.2	15.6 ± 8.5	0.75
Tracheostomy	0 (0.0)	3 (42.9)	0.53
Disposition, *n* (%)			0.71
Home	0 (0.0)	0 (0.0)	
Rehab. hospital	2 (66.7)	0 (0.0)	
Skilled nursing facility (SNF)	0 (0.0)	7 (70.0)	
Hospice	0 (0.0)	2 (20.0)	
Hospital length of stay (days)	31.3 ± 35.2	21.0 ± 13.9	0.67
ICU length of stay	12.7 ± 9.6	14.4 ± 12.3	0.83
Dead, *n* (%)	1 (33.3)	1 (10.0)	0.42

* *p* value is result of *t*-test and chi-squared or Fisher’s exact tests for continuous and categorical variables, respectively

**Table 4:** Multivariable regression analysis for the association between younger men and outcomes compared to older men.

Outcome	* p
Hospital length of stay Younger men compared to older men	0.011
Intensive care unit stay Younger men compared to older men	0.066

* Each row in the table represents a separate multivariable regression analysis adjusted for injury severity.
